# Combustion of lean methane over Co_3_O_4_ catalysts prepared with different cobalt precursors†

**DOI:** 10.1039/c9ra09544f

**Published:** 2020-01-28

**Authors:** Yifan Zheng, Yueqin Yu, Huan Zhou, Wanzhen Huang, Zhiying Pu

**Affiliations:** College of Chemical Engineering, Zhejiang University of Technology Hangzhou 310014 China; Research Center of Analysis and Measurement, Zhejiang University of Technology Hangzhou 310014 China zhiyingpu@zjut.edu.cn

## Abstract

To investigate the effect of catalyst precursors on physicochemical properties and activity of lean methane catalytic combustion, a series of Co_3_O_4_ catalysts were prepared *via* a precipitation method by using four different cobalt precursors: Co(C_2_H_3_O_2_)_2_, Co(NO_3_)_2_, CoCl_2_, and CoSO_4_. The catalysts were characterized by BET, XRD, SEM, Raman, XPS, XRF, O_2_-TPD and H_2_-TPR techniques. It was found that the different types of cobalt precursor had remarkable effects on the surface area, particle size, reducibility and catalytic performance. In contrast, the Co_3_O_4_-Ac catalyst showed a relatively small surface area, but its activity and stability were the highest. XPS, Raman, O_2_-TPD and H_2_-TPR results demonstrated that the superior catalytic performance of Co_3_O_4_-Ac was associated with its higher Co^2+^ concentration, more surface active oxygen species and better reducibility. In addition, the activity of the Co_3_O_4_-S catalyst reduced significantly due to the residual impurity SO_4_^2−^, which could reduce the concentration of surface adsorbed active oxygen species and inhibit oxygen migration.

## Abstract 2

The effects of cobalt precursor on the microstructure, surface properties, reducibility and catalytic performance for methane combustion were investigated.

## Graphical abstract

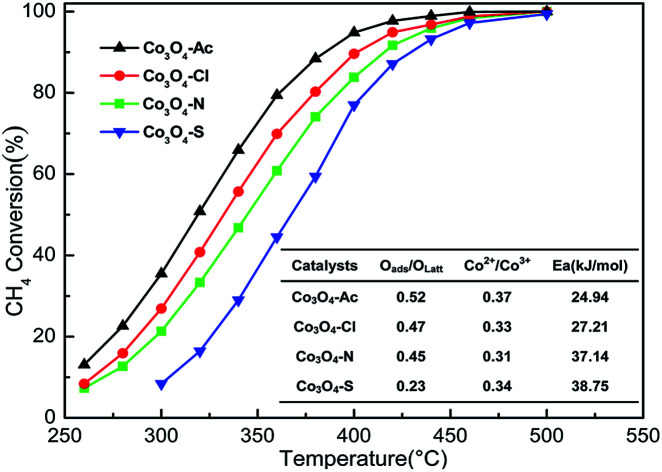

## Introduction

Nowadays, there has been considerable attention focused on energy shortage and environmental protection issues. Recent decades have witnessed increasing requirements of natural gas for many fields owing to its abundant reserves.^1–3^ Nevertheless, the exhaust emission directly releases into the atmosphere because of the incomplete combustion of natural gas, which is a great threat to the environment. Methane, the main component of natural gas, is regarded as the second most damaging greenhouse gas, contributing approximately 20 times more to global warming than carbon dioxide.^4,5^ However, it is difficult for methane to be oxidized due to the strongest C–H bond among hydrocarbons.^6^ The main ways to perform complete oxidation of methane are flame combustion and catalytic combustion. But higher reaction temperature, emission of unburned hydrocarbons and more polluting environmental pollutants (NO_*x*_), as well as lower energy efficiency represent serious disadvantages of flame combustion.^7^ Catalytic combustion is considered to be one of the most efficient and promising technologies for the removal of methane due to its low operating temperature and high efficiency.^8,9^ Thus, it is crucial to develop a catalyst with outstanding catalytic ignition activity and high thermal stability for complete methane combustion.

Supported noble metals catalysts, especially Pd and Pt, have been widely used in complete combustion of methane due to their superior performance.^10–13^ However, the inherent high cost and inferior anti-sintering of noble metals limit their extensive application in the commerce, motivating the investigation of alternative transition metal (Co, Mn, Fe, Cr, Cu, *etc.*) oxides catalysts.^14–17^ Among these candidate metal oxides, cobalt-based oxides are well known because of the structured and electronic properties of spinel type oxides with variable valence states (Co^2+^/Co^3+^) as well as the lower bonding energy of Co–O bonds, which exhibit high activity for catalytic combustion of lean methane.^18^ Therefore, the cobalt-based catalysts have extensive applications in sensors,^19^ lithium-ion batteries,^20^ magnetic materials,^21^ pigments^22^ and solar cells,^23^ which also show high activity for the oxidation of CH_4_, CO and VOCs due to their high redox ability and strong oxygen mobility.^24,25^

It is well known that the catalytic performance of catalysts is closely related to its structure and properties, which strongly depends on the catalyst synthesis method and the types of catalyst precursors.^26,27^ As reported by Li *et al.*, SiO_2_-supported Co catalysts derived from Co(OH)_2_ and Co(NO_3_)_2_ exhibited higher reducibility and CO conversion and C5+ selectivity than those from CoCl_2_ and Co(C_2_H_3_O_2_)_2_.^28^ Jean-Sébastien Girardon and coworkers revealed that the catalyst derived from Co(NO_3_)_2_ showed higher reducibility than from Co(C_2_H_3_O_2_)_2_ and their catalytic activity in FT synthesis depended on the concentration of cobalt metal sites.^29^ However, only few pioneering studies have been reported to explore the influence of different cobalt precursors on the catalytic combustion of methane over Co_3_O_4_ catalysts.

In this work, a series of Co_3_O_4_ catalysts were synthesized by a precipitation method with different cobalt precursors. Multiple techniques including BET, XRD, SEM, Raman, XPS, XRF, O_2_-TPD and H_2_-TPR were applied to characterize the physical and chemical properties. The specific objectives were to evaluate the effect of cobalt precursors for methane combustion.

## Experimental

### Catalyst preparation

A series of Co_3_O_4_ catalysts were prepared by a precipitation method previous published.^30,31^ All chemicals were in analytical grade and used as received without purification. With the preparation of a Co_3_O_4_ sample by precipitating Co(C_2_H_3_O_2_)_2_ solution as an example here, Na_2_CO_3_ aqueous solution (1 mol L^−1^) was added drop by drop into Co(C_2_H_3_O_2_)_2_ solution (0.3 mol L^−1^), accompanied by continual magnetic stirring at 25 °C until the pH reached 9 since the Co_3_O_4_ catalyst prepared at pH = 9.0 showed the best catalytic activity according to our previous work.^32^ The precipitate was immediately vacuum-filtered and washed with deionized water. Afterwards, the precipitate was dried at 105 °C overnight. Finally, all the samples were calcined at 500 °C in static air for 4 h to get the final catalyst, which was named Co_3_O_4_-Ac. All the other Co_3_O_4_ samples were prepared basically as the same procedures by changing the following cobalt precursors: CoCl_2_, Co(NO_3_)_2_ and CoSO_4_. The achieved catalysts were denoted as Co_3_O_4_-Cl, Co_3_O_4_-N and Co_3_O_4_-S, respectively.

### Catalyst characterization

Nitrogen adsorption and desorption isotherms were measured on a micromeritics apparatus (ASAP 2020). All samples were outgassed under vacuum at 300 °C for 5 h prior to analysis. The surface areas of the samples were calculated using the Brunauer–Emmett–Teller (BET) method.

Powder X-ray diffraction (XRD) patterns of the samples were recorded on PANalytical X'Pert PRO X-ray diffractometer using Cu Kα radiation (*λ* = 0.1541 nm) at 40 kV and 40 mA. The patterns were collected with the 2*θ* range from 10 to 80° at the step of 0.03°. The average crystallite sizes of the samples were calculated using the Scherrer equation based on the most intense *hkl* (3 1 1) diffraction peak of Co_3_O_4_.

Scanning electron microscopy (SEM) characterizations were performed on a Hitachi S-4700 instrument operated at 15 kV. The samples were covered with a thin layer (5 nm) of Pt by sputter coating before analysis.

Raman measurements were performed on a LABRAM-HR confocal laser Raman spectrometer using a 514 nm laser source, scanning from 100 to 800 nm. Before measurement, the powder samples were pressed to form disks with a homemade mold.

The content of impurity elements (S) in the investigated catalysts, in the form of pressed discs, was determined with the use of Wavelength-Dispersive XRF spectrometer (ThermoFisher Scientific, ADVANT'X 4200). The X-rays were generated with the Rh anode. For quantitative analysis, the calibration with a series of metallic standards and UniQuant software were used.

X-ray photoelectron spectroscopy (XPS) measurements were performed with a Kratos AXIS UItra DLD spectrometer using the Al X-ray source. The working voltage was 15 kV and the working current was 15 mA. The binding energies were calibrated using C 1s peak of contaminant carbon (284.8 eV) as standard. The spectra were performed with XPSPEAK software (ver. 4.1).

The O_2_ temperature programmed desorption (O_2_-TPD) was conducted on a chemisorption analyzer (AutoChem II 2920), 100 mg of sample was loaded in a quartz reactor, and cleaned in a He flow (30 mL min^−1^) at 200 °C for 30 min, followed by cooling down to 50 °C in the same flow. Afterward, a flow of 21% O_2_/N_2_ at a rate of 30 mL min^−1^ was passed through the sample for 1 h at 50 °C. Finally, the sample was heated to 420 °C at a rate of 10 °C min^−1^ for the desorption of the previous adsorbed oxygen in a flow of He (30 mL min^−1^).

The H_2_ temperature programmed reduction (H_2_-TPR) instrument equipped with a thermal conductivity detector (TCD) was used to investigate the reducibility of the samples. 30 mg of the samples was placed into a quartz tube, reduced in 5% H_2_–Ar gas mixture (40 mL min^−1^) raised to 700 °C with a heat rate of 10 °C min^−1^.

### Catalytic performance evaluation

The activity test for methane combustion was conducted in a fixed-bed quartz tubular reactor (internal diameter = 6 mm) at atmospheric pressure, loaded with 200 mg of the catalyst sieved between 40 and 60 mesh. The temperature inside the reactor was measured by a multipoint K type thermocouple placed in the middle of the catalyst bed. The volume composition of the feed gas was 0.5% CH_4_, 8.0% O_2_ and 91.5% N_2_. The total flow rate of the feed gas is 30 mL min^−1^. The reactants and products were analysed on an Echrom A90 gas chromatograph equipped with hydrogen flame ionization detector (FID) and Ni catalyst convertor. The methane conversion was calculated from the inlet and outlet concentration of CH_4_. Arrhenius equation can be simplified since the composition of reactant gas remains essentially unchanged. Thus the activation energy (*E*_a_, kJ mol^−1^) was evaluated according to the following equations:

1 *r* = *N* × *X*/*W*

2 ln *r* = *E*_a_/*RT* + *C*

where *N*, *W* and *r* are total flow rate of methane (mol s^−1^), the catalyst's weight (g), and reaction rate (mol g^−1^ s^−1^) of methane, respectively; *R* = 8.314 J mol^−1^ K^−1^; *T* is the reaction temperature (K^−1^); *C* is a constant.

## Results and discussion

### Structural and morphological properties

The BET surface areas of Co_3_O_4_ catalyst are listed in Table 1. The *S*_BET_ of Co_3_O_4_-Ac, Co_3_O_4_-Cl, Co_3_O_4_-N and Co_3_O_4_-S are 15.4, 12.2, 25.2 and 25.6 m^2^ g^−1^, respectively. In contrast, the Co_3_O_4_-Ac and Co_3_O_4_-Cl exhibit relatively smaller surface areas than Co_3_O_4_-N and Co_3_O_4_-S. The results indicate that the cobalt precursors greatly affect the physical properties of these catalysts.

**Table 1:** Surface areas, average crystallite size and *I*_192_/*I*_684_ of Co_3_O_4_ catalysts prepared with different cobalt precursors

Catalysts	*S*_BET_ (m^2^ g^−1^)	Crystallite size (nm)	*I*_192_/*I*_684_
Co_3_O_4_-Ac	15.4	46.8	0.18
Co_3_O_4_-Cl	12.2	57.0	0.16
Co_3_O_4_-N	25.2	39.7	0.16
Co_3_O_4_-S	25.6	35.9	0.17

**Fig. 1:**
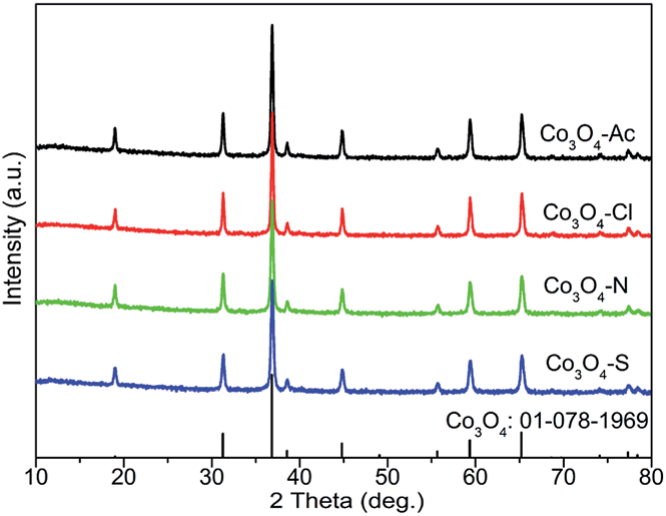
XRD patterns of Co_3_O_4_ catalysts prepared with different cobalt precursors.

XRD technique has been used to identify both the phase composition and the crystallite size of the Co_3_O_4_ samples prepared with different precursors, as displayed in Fig. 1. The peaks at 2*θ* = 19.0, 31.3, 36.8, 38.6, 44.8, 55.7, 59.4 and 65.2° are assigned to the (1 1 1), (2 2 0), (3 1 1), (2 2 2), (4 0 0), (4 2 2), (5 1 1) and (4 4 0) lattice planes of spinel Co_3_O_4_ oxide (ICDD: 01-078-1969).^33^ No any characteristic diffraction peaks for impurities are observed in the XRD patterns, illustrating the complete transition into Co_3_O_4_ or the impurities content is too low to be detected. The average crystallite size of the Co_3_O_4_ samples were estimated from XRD result using the Scherrer equation and listed in Table 1. The crystallite size of Co_3_O_4_-Ac and Co_3_O_4_-Cl catalysts are 46.8 and 57.0 nm, respectively, higher than that of the other two catalysts (39.7 and 35.9 nm), which is likely due to the precursors in the preparation process affecting the nucleation and growth rate of crystallite during precipitation. This is in good agreement with the BET results.

**Fig. 2:**
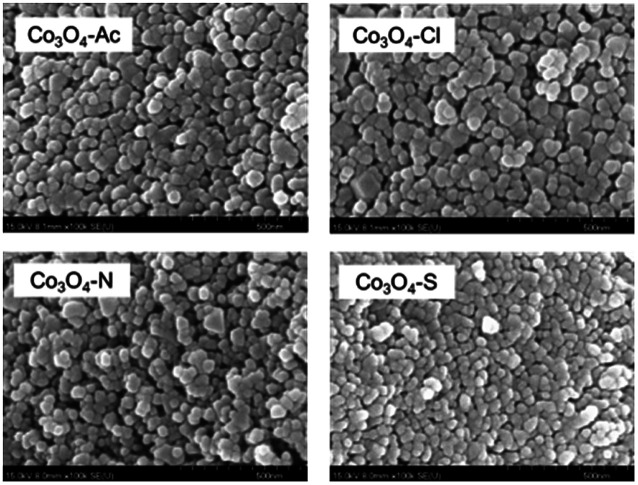
SEM images of Co_3_O_4_ catalysts prepared with different cobalt precursors.

The SEM images of the Co_3_O_4_ catalysts prepared with different cobalt precursors are presented in Fig. 2. Apparently, the catalyst particles have good dispersion and smooth surface with uniform spherical shape. There is almost no agglomeration occurred in Co_3_O_4_ catalysts and a large number of particles are generated in the size range 30 to 60 nm. It can be noticed that the Co_3_O_4_-Ac, Co_3_O_4_-Cl, Co_3_O_4_-N and Co_3_O_4_-S catalysts have the average size about 46, 53, 42 and 37 nm, respectively, measured and calculated by Nano Measurer software. That's to say, the Co_3_O_4_-Ac and Co_3_O_4_-Cl catalysts have relatively larger particle size than other samples. This is in accordance with the XRD and BET results, which further illustrates that the cobalt precursors highly influence the grain sizes and morphologies of Co_3_O_4_ catalysts.

### Surface analyses

**Fig. 3:**
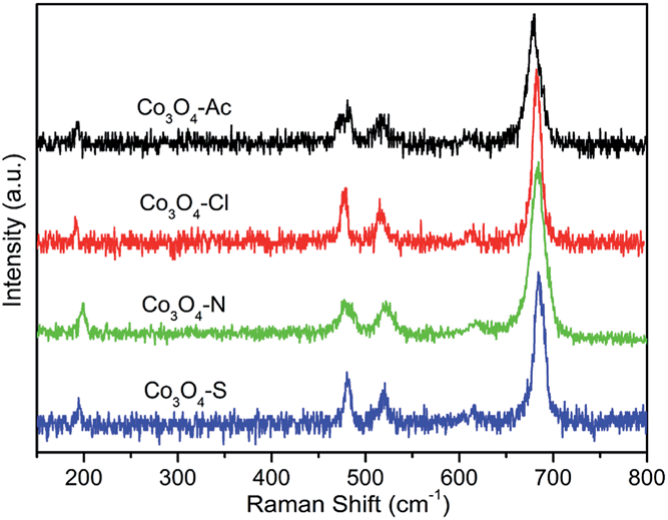
Raman spectra of the Co_3_O_4_ catalysts prepared with different cobalt precursors.

Fig. 3 shows the Raman spectra of the Co_3_O_4_ catalysts. Five vibration modes at 192, 476, 516, 615 and 684 cm^−1^ are observed in the spectra at ambient conditions. The weak band located at 476 cm^−1^ can be assigned to the *E*_g_ symmetry, while the peaks at 516 and 615 cm^−1^ are assigned to the *F*_2g_ symmetry. Furthermore, the band at 192 cm^−1^ is assigned to tetrahedral sites (CoO_4_), corresponding to *F*_2g_ symmetry mode, whereas the band around 684 cm^−1^ is attributed to the characteristics of octahedral sites (CoO_6_) with *A*_1g_ symmetry, respectively.^34,35^ The peak positions are in agreement with those reported for crystalline Co_3_O_4_,^36^ which further confirms the XRD results. According to the literature, Co_3_O_4_ is a transition metal oxide with a spinel structure in which the Co^2+^ ions occupy the tetrahedral sites while the Co^3+^ ions are situated at the octahedral sites due to the phonon symmetries of these Raman bands.^37,38^ Therefore, the intensity ratios of 192 cm^−1^ and 684 cm^−1^ (*I*_192_/*I*_684_) band, indicating the ratio of Co^2+^ and Co^3+^, were calculated and listed in Table 1. It can be clearly seen that *I*_192_/*I*_684_ of Co_3_O_4_-Ac catalyst is higher than the others. That is to say, Co_3_O_4_-Ac catalyst possesses the highest Co^2+^ concentration on the catalyst surface. In addition, it's worth noting that the Raman bands for the highly active Co_3_O_4_-Ac catalyst shift slightly to lower frequencies. It is reported that this phenomenon can be taken as a sensitive indication of a higher concentration of cobalt in low state.^39^ That is to say, Co_3_O_4_-Ac catalyst possesses the highest Co^2+^ concentration on the catalyst surface, which is also further confirmed by the XPS results described below.

**Fig. 4:**
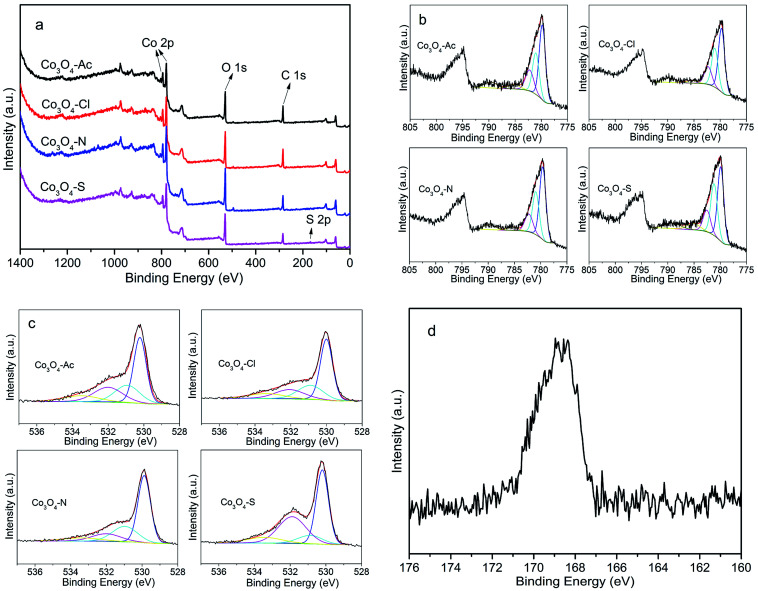
XPS spectra of Co_3_O_4_ catalysts prepared with different cobalt precursors: full scan spectra (a), Co 2p (b), O 1s (c) and S 2p (d).

The surface composition and the oxidation state of the prepared Co_3_O_4_ catalysts were investigated by X-ray photoelectron spectroscopy (XPS) analysis. Fig. 4(a) shows the full scan spectra of Co_3_O_4_ catalysts, in which the Co 2p, O 1s peaks can be observed. The corresponding high-resolution XPS spectra of Co 2p and O 1s spectra of Co_3_O_4_ catalysts prepared with different cobalt precursors are shown in Fig. 4(b) and (c). Generally, Co_3_O_4_ has two types of cobalt ions, containing Co^3+^ in octahedral coordination and Co^2+^ in tetrahedral coordination. As shown in Fig. 4(b), the Co 2p spectra have two major peaks centered at 780.0 (Co 2p_3/2_) and 794.9 eV (Co 2p_1/2_) with a spin–orbit splitting of ∼15.1 eV, indicating the existence of both Co(ii) and Co(iii).^40^ Satellite peak at about 786.0 eV is also a fingerprint for recognition of Co^2+^.^41,42^ The Co 2p_3/2_ and its satellites recorded from the catalysts were deconvoluted into five contributions. Detailed peak deconvolution and the peak assignment were listed in Table 2. The peaks at around 779.8, 779.1 and 782.2 eV can be ascribed to Co^3+^ in octahedral coordination, mixed Co(ii,iii), Co^2+^ in tetrahedral coordination, respectively.^32,43,44^ The area ratios of Co^2+^ and Co^3+^ are calculated and listed in Table 2. As presented in Table 2, the highest ratio of Co^2+^/Co^3+^ is 0.37 for Co_3_O_4_-Ac catalyst, implying that more Co^2+^ appeared on the catalyst surface, which is in accordance with the Raman results. According to the electroneutrality principle, an increase in the Co^2+^ concentration means a rise in the amount of oxygen vacancies,^45^ that is, surface oxygen vacancies are more easily formed on the surface of Co_3_O_4_-Ac nanoparticles. According to the literature, oxygen vacancies not only can activate adsorbed oxygen and provide the lattice sites of oxygen migration, resulting in the formation of highly active electrophilic oxygen species, but also can generate new defective states in the energy band gap of Co_3_O_4_, leading to enhancement of the electronic conductivity of Co_3_O_4_,^46,47^ which can be further confirmed by the high-resolution O 1s XPS spectra.

**Table 2:** XRF and XPS results for the Co_3_O_4_ catalysts prepared with different cobalt precursors

Catalysts	O_ads_/O_Latt_	Co^2+^/Co^3+^	Binding energy of Co 2p_2/3_ (eV)			S content (wt%)
Co_3_O_4_-Ac	0.52	0.37	779.8	781.0	782.2	—
Co_3_O_4_-Cl	0.47	0.33	779.7	781.0	782.3	—
Co_3_O_4_-N	0.45	0.31	779.6	780.9	782.3	—
Co_3_O_4_-S	0.23	0.34	779.9	781.2	782.6	0.324
Attribution	—	—	Co^3+^	Co^3+^; Co^2+^	Co^2+^	—

The O 1s spectra are frequently used to identify the types of oxygen species over the surface of catalysts. The chemical environment of oxygen in metal oxide catalysts plays a critical role in their catalytic properties. The XPS spectra of O 1s over the surface of Co_3_O_4_ catalysts prepared with different cobalt precursors are shown in Fig. 4(c). The asymmetrical O 1s XPS profiles of the catalysts can be deconvoluted into four components. The peaks at 529.9, 530.9, 532.0 and 533.2 eV can be ascribed to the lattice oxygen, surface adsorbed oxygen (O_2_^2−^, O_2_^−^, O^−^), hydroxyl (OH^−^) and/or carbonate species (CO_3_^2−^), as well as adsorbed water, respectively.^48–50^ In general, the electrophilic oxygen species (O_2_^2−^, O_2_^−^, O^−^) are vital for the oxidation of methane.^51^ Therefore, the ratio of the surface adsorbed oxygen and the lattice oxygen (O_ads_/O_Latt_) was calculated and summarized in Table 2. According to early studies, the higher O_ads_/O_Latt_ ratio indicates the richer active oxygen species on the catalysts surface. In addition, it is well known that the adsorbed oxygen species concentration is associated with the oxygen vacancies density. For an oxygen-deficient material, more oxygen vacancies give a higher oxygen species concentration.^52^ As shown in Table 2, the Co_3_O_4_-Ac catalyst has higher O_ads_/O_Latt_ ratio of 0.52 than others, indicating the Co_3_O_4_-Ac catalyst possesses higher adsorbed oxygen species concentration. Therefore, it is reasonable to propose that CH_4_ molecule might have more sufficient contact with the surface active sites of this Co_3_O_4_-Ac catalyst in comparison with other catalysts, thus it could show better methane combustion activity.

For Co_3_O_4_-S catalyst, although the surface Co^2+^ concentration is higher than that of Co_3_O_4_-Cl and Co_3_O_4_-N, the concentration of oxygen species adsorbed on the surface of Co_3_O_4_-S catalysts is the lowest. Considering the deposition of impurity sulfate may cause the catalyst poisoning due to unclean washing,^53^ thus the content of S was measured by XRF (Table 2). It is found the content of S in Co_3_O_4_-S is 0.324 wt%, and no impurity residual is detected in other samples. To further explore the state of S, the S 2p XPS spectrum of the Co_3_O_4_-S sample was performed and presented in Fig. 4(d). It is worth noting the binding energy of S 2p (168.7 eV) is assigned to S(vi) oxidation state,^54^ which demonstrates that there are still non-negligible sulfates on the catalyst surface. These residual impurities SO_4_^2−^ may cover the active sites on the surface of the catalyst, reduce concentration of surface adsorbed active oxygen species, weaken the oxygen mobility, and thus reducing the catalytic performance. It is further confirmed by the catalytic activity of methane combustion.

**Fig. 5:**
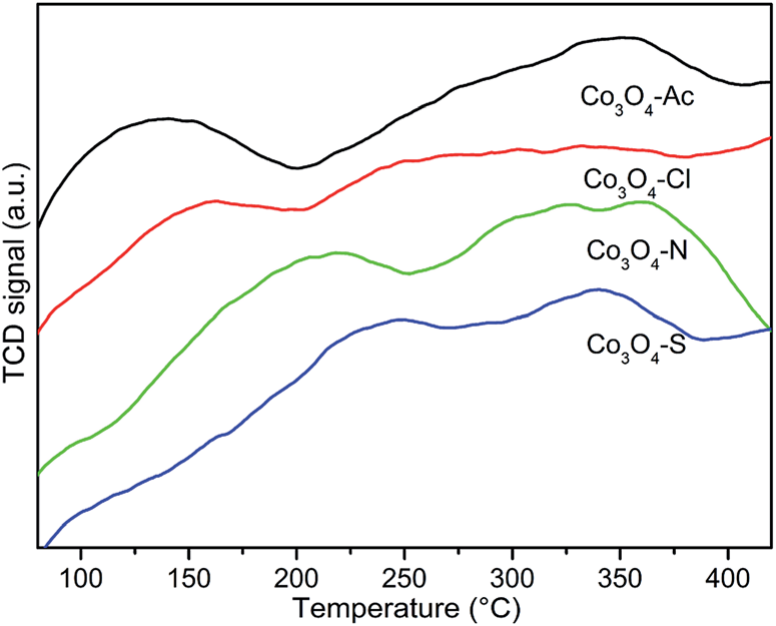
O_2_-TPD patterns of Co_3_O_4_ catalysts prepared with different cobalt precursors.

Fig. 5 shows the O_2_ temperature programmed desorption (O_2_-TPD) profiles. It is reported that the peak below 300 °C is ascribed to the desorption of adsorbed oxygen species such as O_2_^−^, O^−^ in Co_3_O_4_ while the desorption peak of lattice oxygen generally appears above 350 °C.^55^ Additionally, the desorption temperature and the peak intensity are related to both the properties and the amount of surface adsorbed oxygen, which might improve the catalytic activity.^56,57^ Notably, the intensity of desorption peak decreases and the peak gradually shifts to high temperature range follow the sequence of Co_3_O_4_-Ac > Co_3_O_4_-Cl > Co_3_O_4_-N > Co_3_O_4_-S, indicating that the most active Co_3_O_4_-Ac catalyst with lager peak area and lower peak temperature is more preferable for O_2_ dissociative adsorption and easier to produce active oxygen species on the surface.^58^ Therefore, it can be concluded that the amount and the mobility of adsorbed oxygen might be enhanced by changing the cobalt precursors, which is also consistent with the XPS result.

### Reduction properties

**Fig. 6:**
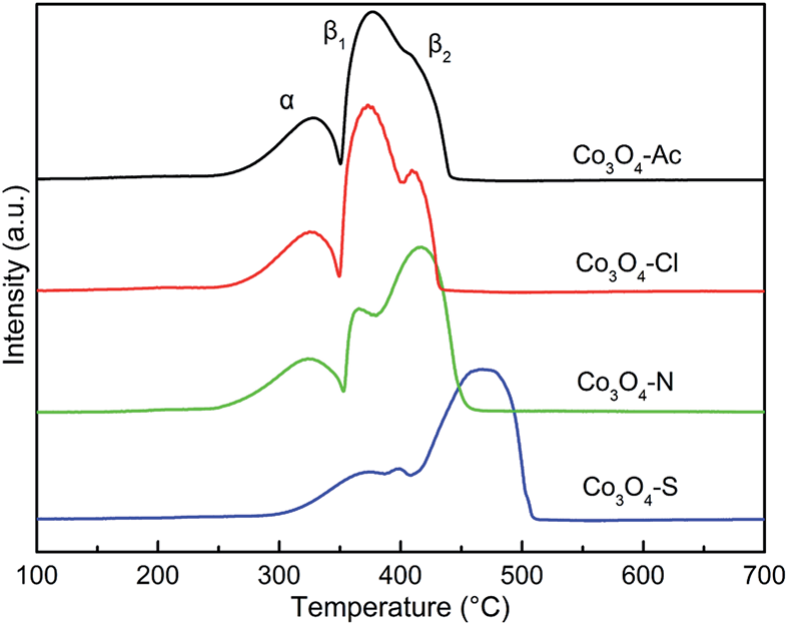
H_2_-TPR patterns of Co_3_O_4_ catalysts prepared with different cobalt precursors.

The H_2_ temperature-programmed reduction measurements (H_2_-TPR) were carried out over the Co_3_O_4_ catalysts prepared with different cobalt precursors to investigate the reduction behavior. Fig. 6 shows the H_2_-TPR profiles of the catalysts. Distinguished from the reduction peak shapes, it is obvious that the change of cobalt precursors has influence on the redox property of the prepared Co_3_O_4_ catalysts. All the Co_3_O_4_ samples display two main reduction peaks at 250–550 °C. The low temperature peak (α) and the high temperature peak (β_1_ and β_2_) are assigned to stepwise the reduction of Co_3_O_4_ to CoO and subsequently CoO to Co,^59,60^ respectively. The α peaks of Co_3_O_4_-Ac, Co_3_O_4_-N and Co_3_O_4_-Cl catalysts are very similar with the lowest reduction temperatures. In contrast, the reduction temperature of α peak for Co_3_O_4_-S catalyst shifts obviously to higher temperature region, implying the Co_3_O_4_-S catalyst is hard to be reduced. It was reported that the hydrogen consumption peak intensity of the poisoning catalyst would be obviously weakened, and the reduction peaks would shift toward the high temperature.^53^ The XPS results mentioned above also show that SO_4_^2−^ exists on the surface of Co_3_O_4_-S catalyst. Therefore, it can be deduced that the reducibility of the Co_3_O_4_-S catalyst is significantly weakened due to the residue SO_4_^2−^ impurity. The TPR profiles were integrated and calibrated with an appropriate hydrogen consumption to quantitatively measure the total hydrogen consumption and the results are shown in Table 3. The theoretical H_2_ consumption is 16.6 mmol g^−1^ when Co^2+^ and Co^3+^ are entirely reduced to metallic Co.^61^ By comparing the four catalysts, it can be found that their actual hydrogen consumption is close to the theoretical one, indicating that Co_3_O_4_ catalysts have been almost completely reduced to Co, except for Co_3_O_4_-S catalyst. Taking into account the factors mentioned above, we can deduce that the less hydrogen consumption of Co_3_O_4_-S is due to the presence of impurities, which is hard to be reduced. This result is in accordance with the composition of the catalysts.

**Table 3:** The results for H_2_-TPR, *T*_50_, *T*_90_, *E*_a_

Catalysts	Total H_2_ consumption (mmol g^−1^)	*T*_50_ (°C)	*T*_90_ (°C)	*E*_a_ (kJ mol^−1^)
Co_3_O_4_-Ac	16.6	318	385	24.94
Co_3_O_4_-Cl	16.2	332	400	27.21
Co_3_O_4_-N	17.0	344	416	37.14
Co_3_O_4_-S	13.8	367	429	38.75

In addition, the high temperature peak β do not take the shape of a single peak, but instead is divided into two different contributions, which confirms the existence of Co^2+^ species with different reducibility. The peak area of β_1_ for the samples follows the sequence of Co_3_O_4_-Ac > Co_3_O_4_-Cl > Co_3_O_4_-N > Co_3_O_4_-S, while the sequence of β_2_ is opposite. The order of β_1_ peaks in Co_3_O_4_-Ac, Co_3_O_4_-Cl and Co_3_O_4_-N catalysts is consistent with that of surface Co^2+^/Co^3+^ in XPS results. In other words, the higher the ratio of Co^2+^/Co^3+^, the stronger the reduction peak of β_1_. Co_3_O_4_-S, as a special case, displays not only the highest reduction temperatures of α and β peak, but also the lowest peak area of β_1_, although its Co^2+^/Co^3+^ concentration is high. It is likely due to the presence of residue SO_4_^2−^ impurity on the surface of the catalyst, which covers the active sites on the surface of the catalyst, resulting in the reduction of reaction activity. The lowest surface adsorbed oxygen species concentration in XPS results also confirms this conclusion. According to the literature, the reduction temperature of surface Co^2+^ is lower than that of bulk Co^2+^, that is to say, surface Co^2+^ is easier to be reduced.^62^ Combined with our previous XPS results, it can be inferred that β_1_ is assigned to the reduction of surface Co^2+^ and β_2_ is assigned to the reduction of bulk Co^2+^. The higher the concentration of Co^2+^/Co^3+^ and adsorbed oxygen species on the catalysts surface, the easier it is to be reduced.

### Catalytic activity

**Fig. 7:**
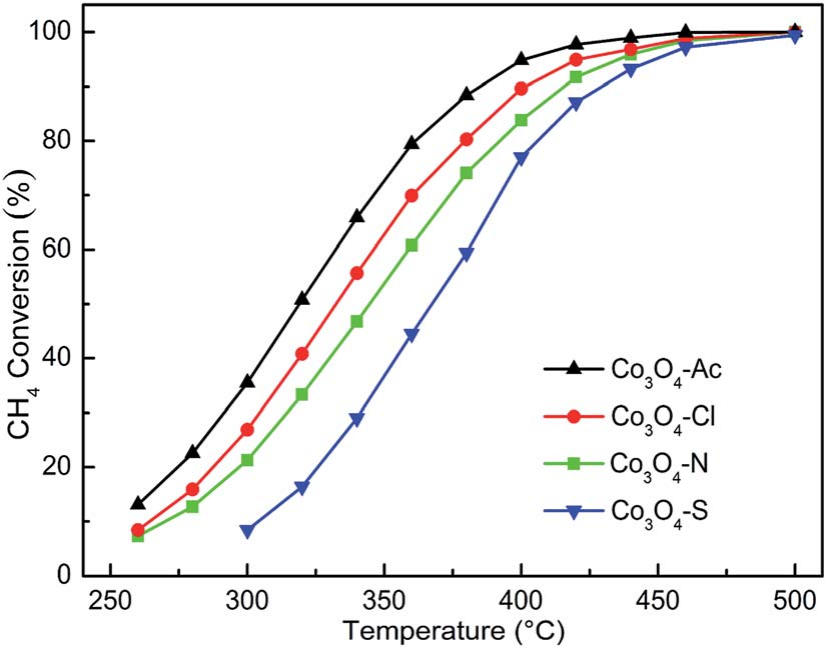
Catalytic activity for methane combustion over Co_3_O_4_ catalysts prepared with different cobalt precursors.

Fig. 7 represents the activity for methane combustion over Co_3_O_4_ catalysts prepared with different cobalt precursors. The catalytic activity of Co_3_O_4_ catalysts decreases in the order of Co_3_O_4_-Ac > Co_3_O_4_-Cl > Co_3_O_4_-N > Co_3_O_4_-S. As shown in Table 3, the characteristic temperatures *T*_50_ and *T*_90_ correspond to the initiation of the oxidation, 50% conversion and 90% conversion of CH_4_. It is clear that Co_3_O_4_-Ac catalyst presents the best activity. In general, catalysts with smaller crystallite size and higher specific surface area expose more active sites and facilitate the catalytic oxidation process. However, no obvious relationship between catalytic activity and surface area was found. The most important thing for the catalyst to enhance the catalytic performance is to decrease the activation energy (*E*_a_) of the reaction, making the catalyst easier to be initiated.^63^ Arrhenius plots for Co_3_O_4_ catalysts are shown in Fig. S1 (ESI†). The *E*_a_ can be obtained from the slope of the linear plot of ln *r versus* 1/*T* according to eqn (2) and the data are listed in Table 3.^64^ It is obvious that the sequence of the *E*_a_ is Co_3_O_4_-Ac < Co_3_O_4_-Cl < Co_3_O_4_-N < Co_3_O_4_-S, which is in well agreement with the activity. The Co_3_O_4_-Ac catalyst possesses the lower activation energy (*E*_a_ is 24.94 kJ mol^−1^) and a better performance with a lower reaction temperature at 318 °C for the 50% methane conversion, while the conversion is only 16% for the Co_3_O_4_-S sample at this temperature, as comparison. The results imply that some other factors play a more decisive role than surface areas to improve catalytic activity.

From the XPS and several other characterization results mentioned above, it can be found the ratio of Co^2+^/Co^3+^ maybe strictly correlated to the performance. Moreover, the higher the concentration of Co^2+^, the higher the concentration of oxygen species adsorbed on the surface and thus improving the catalytic activity. The Co_3_O_4_-Ac catalyst possesses the highest concentration of Co^2+^/Co^3+^ and surface absorbed oxygen which enable the enhancement of its catalytic activity. As a special case, although the surface Co^2+^ concentration of the worst active Co_3_O_4_-S catalyst is higher than that of Co_3_O_4_-Cl and Co_3_O_4_-N catalysts, the concentration of oxygen species adsorbed on the surface of Co_3_O_4_-S catalyst is the lowest due to the influence of residual impurity SO_4_^2−^, which can cause catalyst poisoning and inhibit oxygen migration, thus reducing its methane catalytic performance. Therefore, it can be concluded that the performance of catalytic combustion for lean methane is closely related to the Co^2+^ concentration and surface adsorbed oxygen species, which is consistent with our previous research. The result has proved strongly that by tuning the cobalt precursors, Co_3_O_4_ with optimal redox property and the highest methane combustion activity can be obtained. It indicates that cobalt precursor play a vital role in controlling structural properties of the catalysts, resulting in a significant difference in physicochemical properties and catalytic performance.

### Stability and cyclicity

**Fig. 8:**
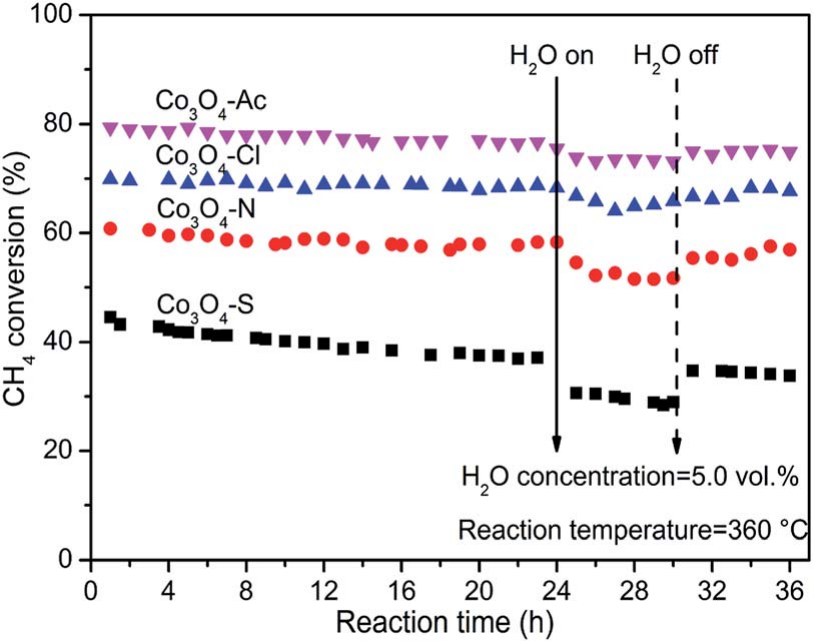
On-stream methane combustion and moisture resistance over Co_3_O_4_ catalysts at 360 °C.

The stability and cyclicity of catalysts play important indicators in the application of catalysts. Thus, the stability, moisture resistance and recycle performance were investigated. The long term stability and moisture were examined at 360 °C and shown in Fig. 8. For each stability test, the methane combustion was carried out using a dry feed for the first 24 h. Afterwards, 5.0% water vapor was added into the reaction feed. After stabilized for 30 min, the first injection started and continued for 6 h. Then the water vapor was taken off until reacting for 36 h. As shown in this figure, methane conversion for Co_3_O_4_-Ac, Co_3_O_4_-Cl and Co_3_O_4_-N catalysts remained, no remarkable loss of catalytic activity is observed in the first 24 h, except for Co_3_O_4_-S catalyst, which shows the worst catalytic performance, decreased from 43% to 37% in the initial period. After 24 h tests, the CH_4_ conversion of Co_3_O_4_-Ac catalyst tends to decline from ∼78% to ∼74% with the moisture (5.0 vol%) adding into the reaction feed for 6 h. After interruption of moisture, the CH_4_ conversion has a recovery in 1 h, stabilized at ∼76%, slightly lower than the initial conversion, which implying that the presence of moisture slightly inhibits the catalytic activity. The moisture resistance (at 360 °C) of all the catalysts decrease as follows: Co_3_O_4_-Ac ≈ Co_3_O_4_-Cl ≈ Co_3_O_4_-N > Co_3_O_4_-S. By comparison, the activity of Co_3_O_4_-S catalyst has rebounded to some extent, but it can be obviously found a pronounced downward within 6 h after interruption of moisture, which may be due to the presence of impurity sulfate. In summary, Co_3_O_4_ catalysts exhibit good long-term stability and moisture resistance in the complete combustion of lean methane if impurities can be effectively avoided.

**Fig. 9:**
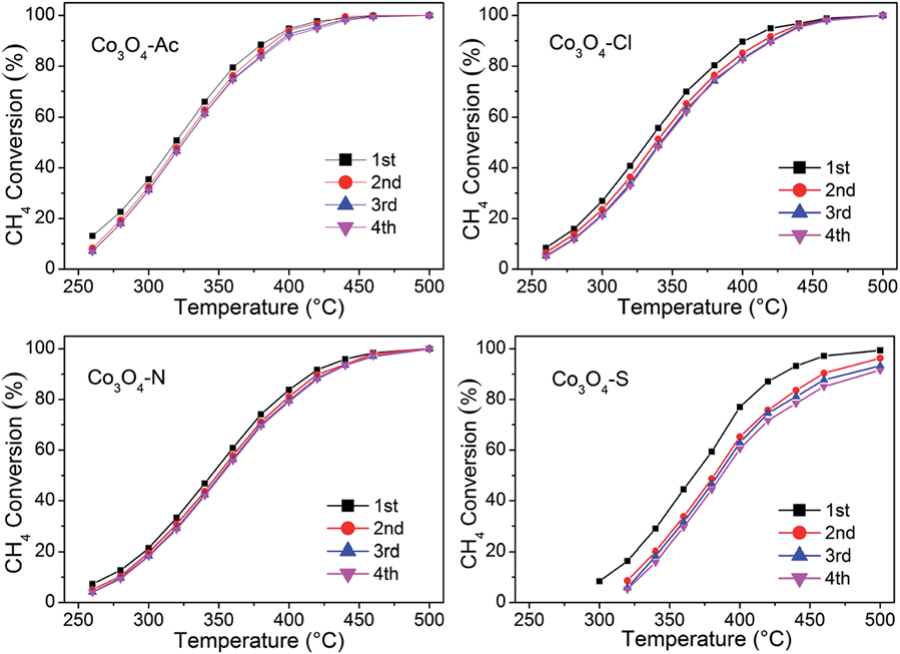
CH_4_ conversion during four consecutive runs for Co_3_O_4_ catalysts prepared with different cobalt precursors.

Fig. 9 shows four consecutive runs curves of methane combustion over Co_3_O_4_ catalysts prepared with different cobalt precursors. It can be seen from the figure that the conversion of the second run curve decreases slightly compared with the fresh sample (first run), but the last three runs curves possess well overlapping, indicating they are effective and stable catalysts for long-term operations, except for Co_3_O_4_-S catalyst with relatively poor recycle performance. Moreover, the used catalysts (after four consecutive runs) were characterized by XRD and Raman techniques, the results are shown in Fig. S2 and S3.† No any other characteristic signals are observed except for the characteristic diffraction peaks and vibration modes of Co_3_O_4_, indicating that the physical–chemical properties of the Co_3_O_4_ catalysts have not changed after the reaction.

## Conclusions

A series of Co_3_O_4_ catalysts have been prepared with a simple precipitation method by changing the cobalt precursors and tested for lean methane combustion. It demonstrated that different cobalt precursors had great influence on the microstructure, surface properties, reducibility, catalytic activity and stability of Co_3_O_4_ catalysts. Co_3_O_4_-Ac catalyst showed the highest performance for lean methane combustion, although it exhibited relatively small BET surface area. The results of Raman and XPS showed that the extraordinary performance was closely related to the concentration of Co^2+^ and surface active oxygen species. As a special case, although the surface Co^2+^ concentration of the worst active Co_3_O_4_-S catalyst was high, the concentration of oxygen species adsorbed on the surface of Co_3_O_4_-S catalyst was the lowest due to the residual impurity SO_4_^2−^, which could cause catalyst poisoning and inhibit oxygen migration. Therefore, the negative effects of residual impurity on catalytic performance should be considered and avoided. Indeed, the information achieved in this study might provide some novel insight for people to design cheaper and more applicable methane combustion catalysts without noble metals for real emission control applications.

## Conflicts of interest

There are no conflicts to declare.

## Supplementary Material

RA-010-C9RA09544F-s001
