# Supplementary material for: Combustion of lean methane over Co3O4 catalysts prepared with different cobalt precursors
Source: RSC Adv. 2020 Jan 28;10(8):4490–8. doi: 10.1039/c9ra09544f (PMC9049175; doi:10.1039/c9ra09544f)
Supplement: RA-010-C9RA09544F-s001 [file RA-010-C9RA09544F-s001.pdf]

Electronic Supplementary Material (ESI) for RSC Advances.
This journal is © The Royal Society of Chemistry 2020

Electronic Supplementary Information

For

Combustion of lean methane over Co_3O_4 catalysts prepared with different cobalt precursors

Yifan Zheng,[ab] Yueqin Yu,[ab] Huan Zhou,[b] Wanzhen Huang[b] and Zhiying Pu*[ab]

[a] *College of Chemical Engineering, Zhejiang University of Technology, Zhejiang University of Technology, Hangzhou 310014, China*

[b] *Research Center of Analysis and Measurement, Zhejiang University of Technology, Hangzhou 310014, China.*

Corresponding author: Zhiying Pu

E-mail address: zhiyingpu@zjut.edu.cn

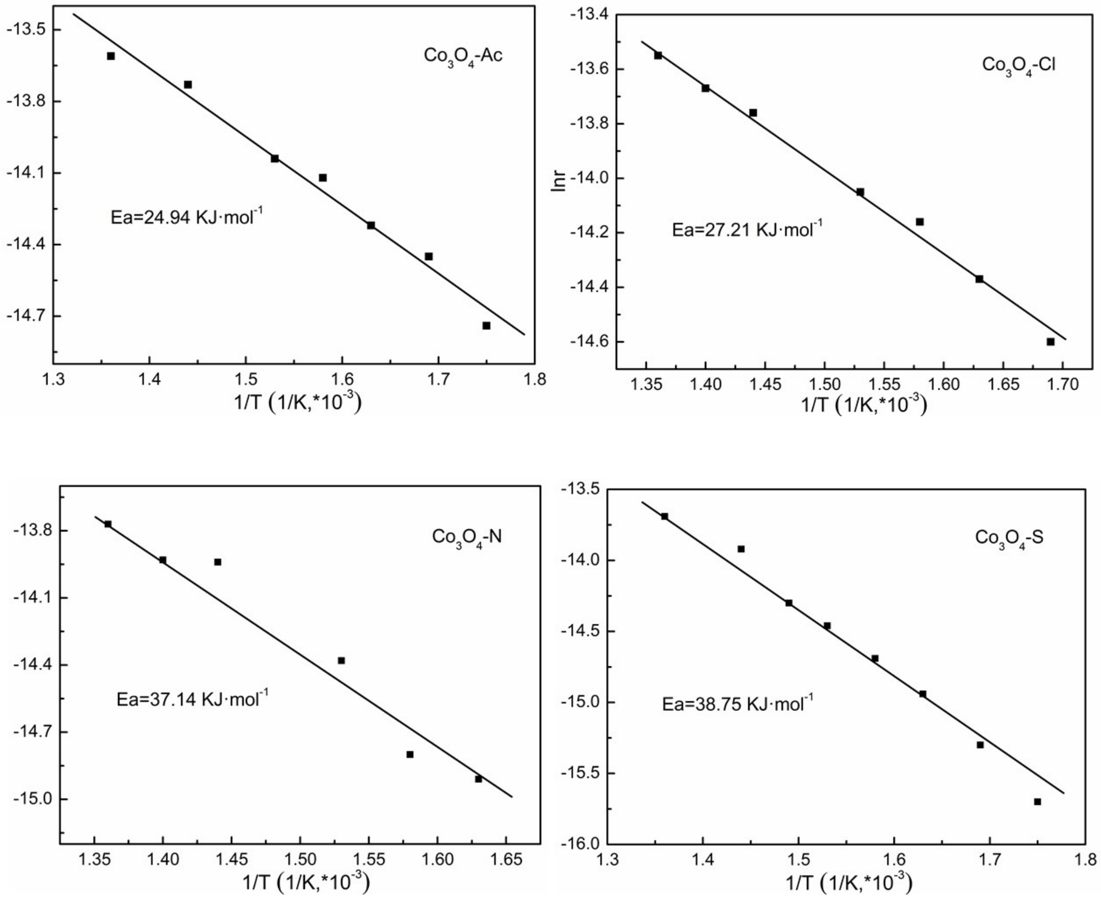

Fig. S1 Arrhenius plots of lnr versus 1/T for Co_3O_4 catalysts prepared with different cobalt precursors

The activation energy (Ea) can be obtained from the slope of the linear plot of lnr versus 1/T. The order of the activation energy for all the catalysts is as follows: Co_3O_4-Ac < Co_3O_4-Cl < Co_3O_4-N < Co_3O_4-S, which is in well agreement with the catalytic activity. Obviously, Co_3O_4-Ac catalyst possesses lower activation energy (Ea is 24.94 $kJ{\cdot}mol^{-1}$) than the other catalysts, suggesting that the catalytic oxidation reaction on Co_3O_4-Ac could be more easily initiated, which could enhance the catalytic activity for methane combustion.

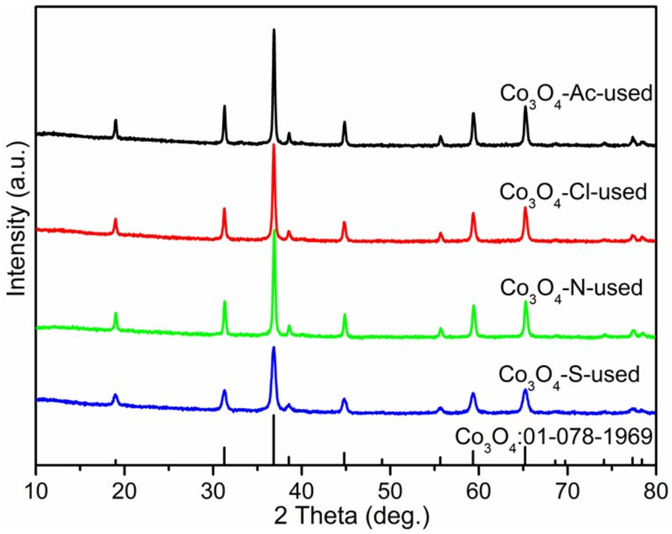

Fig. S2 XRD patterns of used catalysts (after four consecutive runs)

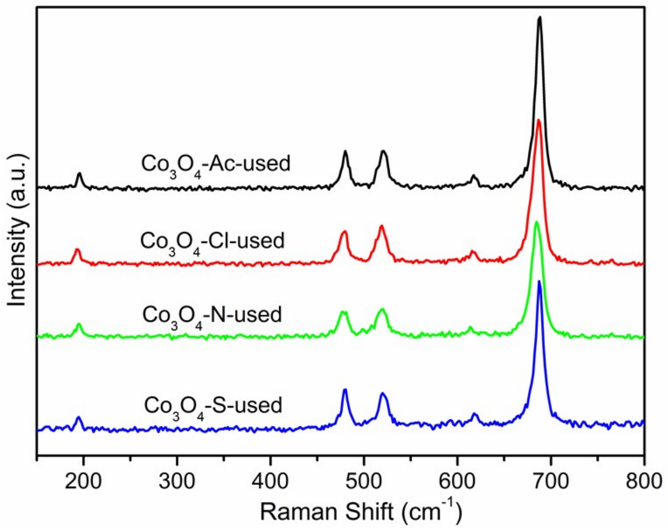

Fig. S3 Raman spectra of used catalysts (after four consecutive runs) in wavenumber range of 150-800 cm^{-1}
